# Using Immediate Response Technology to Gather Electronic Health Data and Promote Telemental Health Among Youth

**DOI:** 10.5334/egems.231

**Published:** 2018-07-31

**Authors:** Tammy Toscos, Maria Carpenter, Michelle Drouin, Amelia Roebuck, Abigail Howard, Mindy Flanagan, Connie Kerrigan

**Affiliations:** 1 Parkview Health, US; 2 Purdue University, Fort Wayne, US

**Keywords:** electronic health data, immediate response technology, mental health, youth, adolescents

## Abstract

**Introduction:**

A sizeable number of youth are currently struggling with anxiety, depression, and suicidal thoughts, yet many will not receive treatment. We sought to better understand if immediate response technology (IRT) could be used to gather mental health care data and educate youth on telemental health (TMH) resources.

**Methods:**

Using an IRT imbedded within an interactive, media-rich school-based presentation, we gathered mental health history and preferences for TMH resources from 2,789 adolescents with a wide range of demographic and psychological characteristics.

**Results:**

More than 80 percent of adolescents satisfied inclusion criteria for survey completion, and responses were statistically comparable across four diverse high school settings. Using Chi-squared analyses, we found that less than 10 percent of adolescents, especially girls and those with high depression/anxiety scores, had previously used TMH resources. After interacting with the IRT, many more (29 percent to 43 percent) expressed willingness to use these resources.

**Discussion:**

The IRT system was effective in gathering mental electronic health data, delivering targeted mental health education, and promoting positive attitudes towards TMH among adolescents.

**Conclusions:**

IRTs and other non-formalized technologies should be explored as cost-effective, easy-to-implement resources for electronic health data gathering and health care education.

## Introduction

Electronic health data, collected both formally and informally, has become a valuable resource in the provision of health care and education. On the formal side, there has been a significant increase in the adoption of electronic health record (EHR) systems, especially since the Health Information Technology for Economic and Clinical Health (HITECH) act was passed in 2009, appropriating approximately $36 billion in funding to hospitals for EHR adoption [1]. This act aligns with the National Strategy for Quality Improvement in Health Care, which emphasizes “the ‘meaningful use’ of certified electronic health record technology to improve patient care and care delivery [2].” Numerous research studies have demonstrated that EHRs can be used effectively to treat and manage patients, and meta-analyses suggest benefits in terms of increased efficiency and quality of health care [3]. However, the use of EHRs is not without issues, including patients’ reticence to share private health information for health care and research purposes [4] and, when used for research, the overrepresentation of sick patients with complete records in EHR databases [5]. Thus, efforts have been made to harness other, less formalized technologies, like crowdsourcing (i.e., large scale Internet sampling) and audience response systems (i.e., audience members respond anonymously to questions on their devices and watch as answers instantaneously populate displays) [6], to gather health data and promote wellness [7,8,9].

Traditional conceptualizations of electronic health data are being challenged as these high-volume, inexpensive data gathering technologies are emerging in health care research [9]. These technologies offer potential windows into the health care characteristics and preferences of targeted populations, including both sick (or at-risk) and healthy individuals. According to a recent study, crowdsourcing has limitations in terms of the similarity of crowdsourced samples to the general population [9]; however, if researchers target their population properly and make appropriate generalizations, it is a potentially invaluable tool for health care research. Meanwhile, audience immediate response technologies (IRTs) have been used in only limited ways in health care research. In a recent iteration of this method, researchers used an IRT to gather data about health behaviors of undergraduate students enrolled in a Health Behaviors course [8]. Through the interactive system prompts and ensuing discussions, researchers gathered student health data and compared it to state and national statistics. Ultimately, this resulted in more positive attitudes and actions towards health-behavior change among participants [8]. IRTs have also been used by other researchers to promote healthy decision making about alcohol abuse [7]. According to LeGreco et al.’s (2010) IRT model, experiences from one’s environment can be used to create meaningful learning moments, which, when filtered through the IRT and accompanying dialogue, can help create a reconstructed, social-based reality [7]. In this study, as students responded anonymously to questions about alcohol-related behaviors, responses were displayed on a screen as bar graphs or charts to establish social norms around alcohol activity. Through viewing these aggregated responses, misperceptions about the high prevalence of dangerous alcohol consumption behavior could be examined and potentially adjusted to a more accurate representation of this behavior. Therefore, IRTs allow for social learning [10] and the recalibration of participants’ perspectives based on displayed social norms.

Gathering electronic health data through these less formalized methods might be especially useful for reaching populations characterized by low engagement with traditional health care systems, such as adolescents. Adolescents (aged 13 to 17) are among the most prolific users of mobile technologies: 73 percent of teens have smartphone access, and 76 percent use social media [11]. Because of the high rates of mobile technology use in this age cohort, IRTs might offer a unique opportunity for data gathering through an easy-to-use and familiar communication medium.

One of the prominent health care issues facing adolescents is psychological distress. Most mental health disorders develop before age 24, and previous research suggests that nearly 20 percent of adolescents are currently experiencing a mental health disorder [12,13,14,15]. Yet, only 39 percent of adolescents suffering with depression, 18 percent with anxiety, and 30–45 percent with suicidality will receive traditional face-to-face therapy [16]. Moreover, many of these mental health disorders will be lifelong and characterized by relapse [14,15,17,18]. Given the high costs associated with mental illness, linking adolescents to prevention and treatment is a major public health concern.

Unfortunately, there are a number of pragmatic barriers that prevent adolescents from receiving mental health treatment, including, but not limited to, a lack of insurance reimbursement and health coverage, high cost, few mental health specialists offering adolescent services, stigma, and parental misunderstanding [19,20,21,22]. Recently, the U.S. Department of Health and Human Services has called for meaningful research on telehealth technology to help fill the ever increasing gap in coverage that many populations are facing [23]. These web and computer-based treatments may help some youth who would not otherwise receive treatment.

Although research shows that (TMH) resources are not yet widely used, youth use TMH resources more than any other age cohort [24,25]. More importantly, existing research suggests that TMH resources are effective in treating depression [26,27,28,29,30], anxiety [31,32,33], eating disorders [34,35], and substance abuse disorders [36,37,38,39] in adolescents. Evidence also supports youth and family satisfaction with TMH services [40,41,42]. In fact, some studies have found that youth prefer Internet-based care to face-to-face care and share more information via technology than they do in person [40,43]. Qualitative research shows that adolescents find TMH helpful, feel a sense of personal choice, and generally like the technology [24]. Other benefits to TMH include 24-hour availability, the ability to reach a broad, diverse audience across a large geographic area, lower cost, and user anonymity [24,25]. In short, TMH resources might provide effective support for mental health issues for this population, filling an oft-cited gap in mental health provision for at-risk youth.

The aims of the present research were to: (1) evaluate the practicability of using an IRT to gather electronic mental health care data from a diverse sample of adolescents (both at-risk and not at-risk) while (2) simultaneously leveraging this technology to deliver education and create positive perceptions about TMH among participants. In line with LeGreco et al.’s 2010 IRT model [7], we expected these experiences to result in reconstructed, social-norm based perceptions of TMH, as measured by their reported willingness to engage with these resources.

## Methods

### Participants

The original sample included 3,412 high school students. However, 168 students who did not complete any questions after the warmup, 434 students who abandoned the survey before the 30th question, and 21 students who responded ‘prefer not to answer’ or did not respond at all for 80 percent or more of the questions were removed. The final sample included 2,789 high school students in grades 9, 10, 11, and 12 (1,442 females, 1,266 males, and 81 who responded “other”) who were enrolled at one of four high schools in different school districts in Northeast Indiana during Spring 2017. Two of the schools were in suburban settings, one was rural, and one was urban, and the percentage of students receiving free or reduced lunch ranged from 32 percent (School A) to 63 percent (School D). Participants’ mean age was 16.1 (*SD* = 1.2), and 63 percent were Non-Hispanic white (see Table 1 for demographic characteristics). To establish the comparability of our final sample to our original sample, subjects who were kept in the analysis were compared to the subjects who were dropped for differences in survey responses. Subjects who were dropped were significantly less likely to be female, non-Hispanic white, and to answer ‘No’ to the suicidality questions (*p* < .05). Additionally, subjects who were dropped had higher scores for PHQ4 items 3 and 4 (depression) but lower scores for item 1 (anxiety); there was a trend (*p* = 0.051) towards a higher proportion with moderate PHQ4 level, but the average scores were not significantly different. Finally, although there was a statistically significant difference in age (*p* = 0.008), the median ages in both sets were 16 and the mean ages were different by only 0.15 years.

**Table 1:** Demographic and mental health characteristics of high school students (*n* = 2,789).

	Full sample *N* (%)

Gender	
Female	1442 (52%)
Male	1266 (45%)
Other	81 (3%)
Race/Ethnicity	
Non-Hispanic White	1746 (63%)
Black	342 (12%)
Other	701 (25%)
PHQ Total Score moderate or severe	590 (21%)
PHQ Anxiety moderate or severe	853 (31%)
PHQ Depression moderate or severe	639 (23%)
Depressive symptoms^a^	914 (33%)
Contemplated suicide^a^	414 (15%)
Prior visit MHP	669 (24%)

*Note*: ^a^In the past year. MHP = Mental Health Provider. Moderate or severe is ≥6 for PHQ total score and ≥3 for PHQ anxiety and depression subscales.

### Data Collection

This study employed a passive consent or “opt-out” consent process. A team of research scientists at a local hospital worked with four partnering schools to ensure that parents were well-informed of the research study and had ample opportunity to opt-out their child(ren). We required schools to communicate a minimum of one time with parents at least two weeks in advance of the survey event date. Each school had a protocol in place for the passive consent process, as they had used this method for other activities.

The IRT survey events occurred during school assemblies in February and March 2017 in the high school’s auditorium or gymnasium. The survey was administered using proprietary polling software on a secure Wi-Fi network. These events were engineered by a contracted company, vetted by the Parkview Health Legal Department and obtained approved security clearance to ensure that the data were secure. All of the content of the survey and event was written, designed and approved by the multi-disciplinary research study team. The events were interactive in that students were shown pre-recorded video content and asked survey questions that they could answer via their personal or school-issued, Wi-Fi enabled electronic device, such as a laptop, tablet, or smartphone.

As students assembled into the school’s auditorium or gymnasium, they were directed by an emcee to connect their smartphones, tablets, or laptops to the secure, private Wi-Fi network. Through scripted instructions, the emcee communicated the goals of the event and also provided participants assurances of the anonymity of their responses. Once connected to the private network on their individual device, students completed an age-appropriate consent/assent process, marking the beginning of the quasi-experimental data gathering. Participants who assented/consented were then asked to respond to survey questions, embedded within a media-rich presentation, which was used to explain and highlight (through high-fidelity videos) some of the most common TMH resources for youth (see Appendix A). The videos contained education on different types of life stressors and teens discussing their experience with stress in a testimonial style. At the conclusion of each video segment, a teen moderator in the video read the related survey prompts while the questions were pushed to the participants devices to gather responses. Therefore, the one-hour program was comprised of short, pre-recorded videos (30.0 percent), survey questions asked through pre-recorded video (41.7 percent) and variable time for student assembly, participant registration and the welcome/conclusion given by the live emcee (28.3 percent). Aside from the mental health questions, all aggregate survey responses were displayed on large screens, which instantly populated figures as students answered questions. These displays served as the dialogue and social norms surrounding TMH resources. All procedures were approved by the Parkview Health Institutional Review Board.

### Measures

The 35-question survey assessed high school students’ mental health as well as their experiences with and preferences for TMH technologies. The survey began with demographic questions (i.e., age, gender, and race/ethnicity) and had the following additional measures.

*Depression and Anxiety* were measured using the Patient Health Questionnaire (PHQ-4) [44], a validated, ultra-brief measure of depression and anxiety [44,45,46] that has been found to be a valid tool in the mass screening of young adults [47]. Students responded on a 4-point Likert scale (0 = *not at all*, 3 = *nearly every day*) about how often in the last two weeks they had experienced anxiety (items 1 and 2) and depression (items 3 and 4) symptoms. We computed scores for the subscales (anxiety Cronbach’s α = .82; depression Cronbach’s α = .76) and also a general PHQ score reflecting the combined symptoms of Depression/Anxiety (Cronbach’s α = .82). According to the scale parameters, total scores of 0–5 are classified as normal to mild, and total scores of 6–12 are classified as moderate to severe in their symptomology.

*Contemplated suicide* was measured with one question from the Youth Risk Behavior Surveillance System (YRBSS) survey [48,49], which is a validated measure of recent and lifetime suicidal thoughts and behaviors in adolescents [50]. Participants were asked whether in the last 12 months “did you ever seriously consider attempting suicide?” with response options of *yes* or *no*.

*Depressive symptoms* in past year was measured with one question from the YRBSS survey [48,49]. Participants were asked whether in the last 12 months “did you ever feel so sad or hopeless almost every day for two weeks or more in a row that you stopped doing some usual activities?” with response options of *yes* or *no*.

*Prior experience with a mental health professional* was assessed in one question: “Have you ever been to a mental health professional (for example: psychiatrist, psychologist, therapist, or counselor)?” Participants responded on a 4-point categorical scale (1 = *yes, and it was helpful*; 2 = *yes, but it was not helpful*; 3 = *I don’t know*; 4 = *no*). For ease of comparison, we collapsed these responses into a binary variable (1 = *yes*; 0 = *no* or *I don’t know*).

*Previous use of telemental health resource*s was assessed with three questions related to their past use of TMH applications or websites. Video demonstrations of existing TMH technologies were included as examples throughout the survey, before students were questioned about their use of these resources. Participants responded about their previous use of *anonymous one-to-one chats, online therapists or counselors*, and *self-help resources*. For each of these questions, students were given categorical choices ranging from “*yes, and it was helpful*” to “*prefer not to answer*.” For ease of analysis, these categories were collapsed into a binary variable (1 = *yes*; 0 = *no* or any other answer, including *I don’t know*).

*Willingness to use telemental health resources* was measured with three questions assessing willingness to engage with *anonymous online chat*, an *online therapist or counselor*, and *self-help resources*. Participants responded on a 5-point Likert scale (1 = *yes, definitely*; 2 = *yes, probably*; 3 = *maybe, I don’t know*; 4 = *no, probably not*; 5 = *no, definitely not*). For ease of analyses, these categories were collapsed into three scale points (1 = *yes*; 2 = *maybe*; and 3 = *no*, including *I don’t know*).

### Data Analysis

There was statistical consistency in survey responses across the four schools; thus, results across the schools were combined. The survey was designed so that students could decline to answer questions. To maintain representativeness in the sample, those participants who provided partial data were retained; thus, analyses were conducted with pairwise deletions. We conducted all analyses with IBM SPSS 24, using chi-squared tests to compare categorical variables.

## Results

Overall, 81.7 percent of those who logged into the IRT provided valid responses for at least 20 percent of the questions and made it through 94 percent of the survey. Of the final sample, a sizeable number reported psychological distress: 33 percent of students reported feeling so sad or hopeless almost every day for two weeks or more in a row that they stopped doing some usual activities, and 15 percent of students had contemplated suicide in the past year. Additionally, almost one fourth (24 percent) had previously seen a mental health professional in a traditional, face-to-face setting. See Table 1 for results.

With regard to previous use of TMH resources, 7 percent had used anonymous online chat and self-help resources for mental health support (see Table 2). Meanwhile, only 3 percent reported using an online therapist. Girls were significantly more likely than boys to have used anonymous online chat and self-help resources (all *p*s < .001). Additionally, those who reported moderate to high levels of depression/anxiety were significantly more likely than those with low levels of depression/anxiety to have used anonymous online chat and self-help resources (all *p*s < .001).

**Table 2:** Frequency of adolescents’ previous use of TMH resources (*n* = 2,789) and results from chi-square tests for differences by gender and PHQ total score in TMH use.

	Yes, *N* (%)	*X*^2^

*Anonymous chat*		
All	189 (7%)	
Female	113 (8%)	*X*^2^ = 12.5, *p* < .001
Male	58 (5%)	
Dep/Anx low/none	101 (5%)	*X*^2^ = 81.4, *p* < .001
Dep/Anx moderate/high	88 (16%)	
*Online therapist*		
All	92 (3%)	
Female	46 (3%)	*X*^2^ = 0.1, *p* = 0.748
Male	37 (3%)	
Dep/Anx low/none	65 (3%)	*X*^2^ = 4.0, *p* = 0.047
Dep/Anx moderate/high	27 (5%)	
*Self-help (used an app/website)*		
All	191 (7%)	
Female	133 (10%)	*X*^2^ = 36.6, *p* < .001
Male	44 (4%)	
Dep/Anx low/none	108 (5%)	*X*^2^ = 61.1, *p* < .001
Dep/Anx moderate/high	83 (15%)	

*Note*: Chi-square tests for association were conducted for previous use of three TMH resources for females versus males and depression/anxiety low/none versus moderate/high.

More students expressed willingness to try these TMH resources than reported previous use of them. Students were most willing to try self-help resources (43 percent said yes or maybe), followed by an online therapist (40 percent said yes or maybe), and anonymous online chat (29 percent said yes or maybe; see Table 3). Akin to the previous use statistics, adolescent girls were significantly more likely than boys to indicate a willingness to try all three types of TMH resources (all *p*s < .01), and those with higher depression/anxiety scores were significantly more likely than those with lower depression/anxiety scores to be willing to try each of these TMH resources (all *p*s < .001).

**Table 3:** Frequency of adolescents’ willingness to use TMH resources (*n* = 2,789) and results from chi-square tests for differences by gender and PHQ total score in willingness to use TMH.

	Yes	Maybe	No	*X*^2^

*Anonymous chat*				
All	274 (10%)	501 (19%)	1783 (67%)	
Female	160 (12%)	305 (22%)	872 (64%)	*X*^2^ = 64.1, *p* < .001
Male	101 (8%)	184 (15%)	865 (72%)	
Dep/Anx low/none	178 (9%)	358 (17%)	1478 (71%)	*X*^2^ = 67.7, *p* < .001
Dep/Anx moderate/high	96 (17%)	143 (25%)	305 (54%)	
*Online therapist*				
All	408 (15%)	689 (25%)	1526 (56%)	
Female	228 (16%)	360 (25%)	799 (56%)	*X*^2^ = 18.2, *p* = 0.003
Male	164 (13%)	308 (25%)	691 (57%)	
Dep/Anx low/none	286 (13%)	541 (25%)	1239 (58%)	*X*^2^ = 24.2, *p* < .001
Dep/Anx moderate/high	122 (21%)	148 (26%)	287 (50%)	
*Self-help (used an app/website)*				
All	524 (20%)	607 (23%)	1476 (56%)	
Female	337 (24%)	387 (28%)	637 (46%)	*X*^2^ = 135.8, *p* < .001
Male	171 (14%)	207 (17%)	792 (66%)	
Dep/Anx low/none	365 (17%)	465 (22%)	1218 (58%)	*X*^2^ = 50.9, *p* < .001
Dep/Anx moderate/high	159 (28%)	142 (25%)	258 (46%)	

*Note*: Chi-square tests for association were conducted for willingness to use three TMH resources for females versus males and depression/anxiety low/none versus moderate/high.

## Discussion

Our first research aim was to examine the practicability of collecting mental health data using an IRT within diverse groups of adolescents. Our high rates of engagement (>80 percent) and the statistical consistency in survey responses across the four schools suggest that IRTs can be used effectively for mental health data collection and education among adolescents in diverse geographic and demographic settings. That said, the non-completers were significantly different from the survey completers on some key attributes. More specifically, they were more likely to be male, non-white, and have higher rates of reported suicidal ideation and depression. Therefore, as with crowdsourcing [9], gathering health data using IRTs does appear to have some sample limitations, at least among adolescents.

With regard to the mental health data we were able to collect, 33 percent of our adolescents experienced depression and 15 percent contemplated suicide in the past year. These results align with previous research that indicates 20 to 40 percent of adolescents are currently struggling with anxiety, depression, and/or suicidal thoughts [12,13,14,15,16] and further emphasize the need to link youth with mental health services. At face value, these results support national norms on the prevalence of psychological distress among adolescents; however, the practical implication of these results is much greater. Specifically, these results show that IRTs can be used to gather baseline mental health data that can be used to develop and evaluate the efficacy of targeted local and regional interventions for at-risk adolescents. The use of IRTs in this way provides a model for the use of non-formalized mechanisms in health data gathering, extending the application and utility of electronic health data outside of traditional hospital settings.

With regard to the effectiveness of the IRT in delivering mental health education and generating positive perceptions about TMH, our results suggest that the IRT system was effective in both areas. Although few adolescents (less than 10 percent) had previously used the highlighted TMH resources, after educating teens on these resources using high-fidelity videos, approximately one third (29 percent to 43 percent) expressed some willingness to engage with different types of TMH. According to LeGreco et al.’s (2010) IRT model, the IRT system provides a unique opportunity for evaluating personal experience in the context of social norms [7]. In this case, we believe that the IRT was useful in reducing stigma surrounding mental health care, allowing participants to develop new social-based norms about TMH resources based on immediate feedback from peers. Notably, willingness to engage with these resources was especially high for girls and those who scored high on the depression/anxiety screen; thus, the IRT was effective in promoting positive attitudes for TMH among *at-risk* adolescents. Leveraging electronic health data collection in this way, targeting at-risk adolescents who may not already be in the health care system, may provide a viable route to early education and intervention for this vulnerable population. Moreover, the low cost and ease of use of IRTs makes these technologies particularly useful for widespread implementation.

## Conclusion

Although the IRT model suggests that IRTs enable dialogue and social learning, we did not contrast the IRT to traditional survey methods, so we cannot conclude that the social norming provided by the activity was an integral component in shaping positive attitudes towards TMH. We look to future research to contrast these methods explicitly. Additionally, our completer sample was different from our original sample on some key characteristics, which limits the generalizability of our findings. Future iterations of IRT research should devise methods to engage these non-completers. Finally, willingness to use TMH was assessed, which might not translate to actual use. Future studies might assess actual TMH use to examine the extent to which behavioral intentions align with behavior change in this population [51].

Overall, our IRT method was effective in collecting mental health care data and providing mental health care education to adolescents from diverse backgrounds within the context of a multi-media school-based presentation. This study adds to a small but growing body of research supporting non-formalized methods of electronic health data gathering and health care education. The ease of use and low cost of these types of data gathering technologies make them especially appealing for future health care research and education; therefore, time and resources should be invested in developing ways to harness these new technologies for widespread health care reform.

## Additional File

The additional file for this article can be found as follows:

10.5334/egems.231.s1 Appendix A Telemental health resources showcased in survey.
