# Supplementary material for: Using Immediate Response Technology to Gather Electronic Health Data and Promote Telemental Health Among Youth
Source: EGEMS (Wash DC). 2018 Jul 31;6(1):19. doi: 10.5334/egems.231 (PMC6078147; doi:10.5334/egems.231)
Supplement: Appendix A — Telemental health resources showcased in survey. [file egems-6-1-231-s1.pdf]

Appendix A.

Telemental health resources showcased in survey:

Anonymous Chat with Trained Non-professionals

- 7 Cups of Tea (www.7cups.com)
- Crisis Text Line (www.crisistextline.org)
- Lifeline Crisis Chat (www.crisischat.org)

Online Therapist Resources (provided by licensed mental health professionals)

- Talkspace (www.talkspace.com)
- BetterHelp (www.betterhelp.com)

Self-help Resources

- Pacifica (www.thinkpacifica.com)
- Now Matters Now (www.nowmattersnow.org)
